# Supplementary material for: Identification and Expression Patterns of WOX Transcription Factors under Abiotic Stresses in Pinus massoniana
Source: Int J Mol Sci. 2024 Jan 28;25(3):1627. doi: 10.3390/ijms25031627 (PMC10855728; doi:10.3390/ijms25031627)
Supplement: Supplementary file 1 [file ijms-25-01627-s001.zip › ijms-2777574-supplementary.pdf]

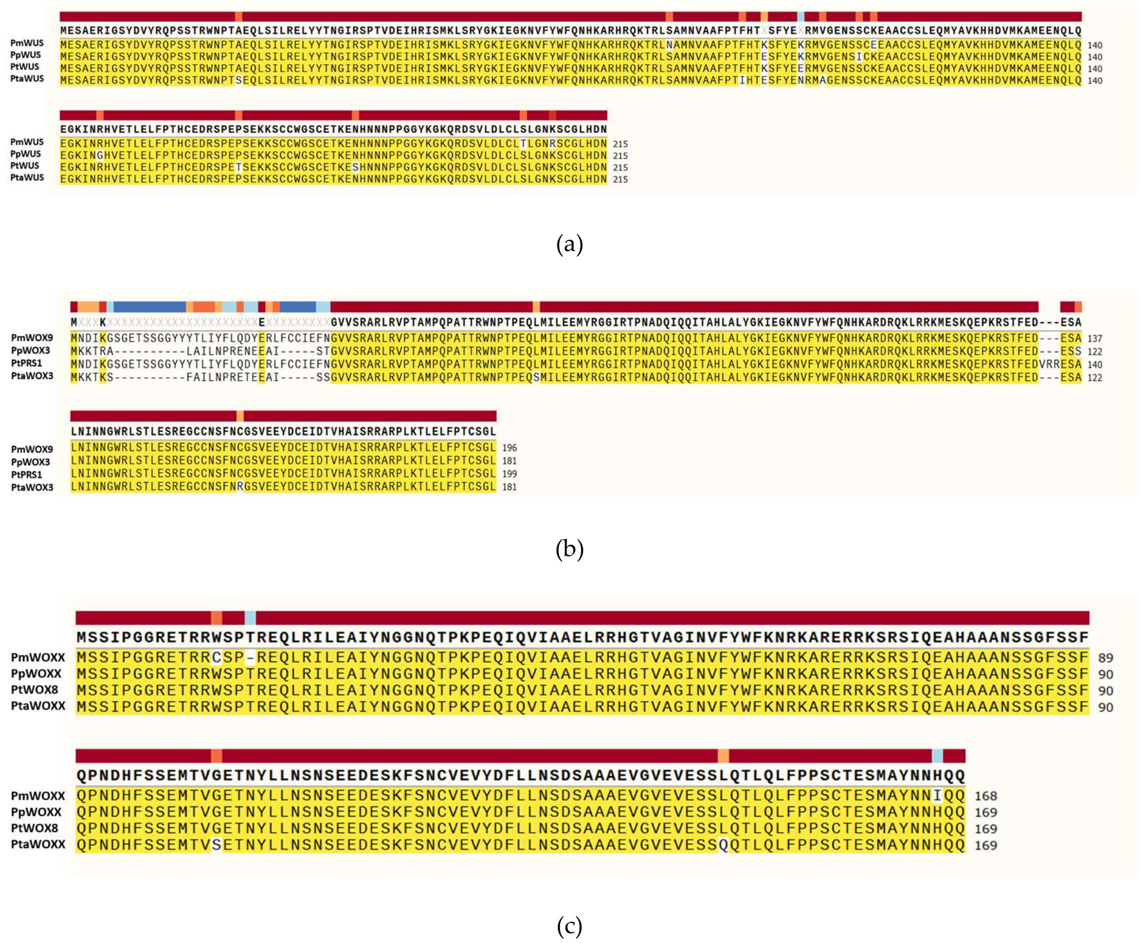

Figure S1. Homologous protein sequence alignments of *PmWOX9* (a) *PmWOXX* (b) and *PmWUS* (c) among *Pinus massoniana,* Pm; *Pinus pinaster*, Pp; *Pinus taeda*, Pta; *Pinus tabulaeformis*, Pt.

Table S1 Sequences of primers

Gene ID	Sequences
TUA-F	CAAACTTGGTCCCGTATCCTC
TUA-R	CACAGAAAGCTGCTCATGGTAA
PmWOX1q-F	CTACCTGTGTCTGGTCAAGGATTTA
PmWOX1q-R	TCTCTTTGCACGAGCTTTACGATTT
PmWOX2q-F	GCCTTCTTCAGTGCCTACTCTGCTT
PmWOX2q-R	TTCCATTGTTATCTCCTTTATTTTT
PmWOX3q-F	GAACCACAAAGCCAGAGAAAGACAG
PmWOX3q-R	GTTAGCAAGGACGTTGAAGTGAACA
PmWOX4q-F	GTTATTGAGACAGAGTAGTGTTGCT
PmWOX4q-R	TGTATTGCTATTTTCCATAGGTTTA
PmWOX9-F	ATGAATGATATAAAGGGATCAGGTG
PmWOX9-R	CTATAAGCCACTGCATGTTGGAAA
PmWOXX-F	ATGAGTTCGATTCCCGGCG
PmWOXX-R	TCACTGTTGATGGTTGTTATAAGCC
PmWUS-F	ATGGAATCTGCCGAGCGAATTGGGA
PmWUS-R	TCAGTTATCATGCAAACCACAGCT
WOX1-BD-F	*atggccatggaggccgaattcATGGAGCCCGAGGATGCTG*
WOX1-BD-R	*ccgctgcaggtcgacggatccTCACCTCAAAAGAGTGTAGCTTTCA*
WOX2-BD-F	*atggccatggaggccgaattcATGCCGTCGGTCACACAGG*
WOX2-BD-R	*ccgctgcaggtcgacggatccTTAAGTTCCCCGATATGGATCTCC*
WOX3-BD-F	*atggccatggaggccgaattcATGGAAGCCAAAATGGCAAA*
WOX3-BD-R	*ccgctgcaggtcgacggatccTCATGTCATGGAATTCAAGAAGGA*
WOX4-BD-F	*atggccatggaggccgaattcATGACGATGGAGATGAATTCTCCA*
WOX4-BD-R	*ccgctgcaggtcgacggatccTTAGGGGAATCTTTTAATTGGATCA*

Table S2 The protein sequences of *PmWOXs*

>PmWOX1

MEPEDAGADKNTGMDNLPHIRLPYQVMTTSQLETLRRQICVYSTICSQLVEMHKAMSQQ
ASTSRERMLYDLPVSGQGFRPSARQRWAPSQTQLHILERLYDHGNCTPNKKKIKEITSELS
QHGPISETNVYNWFQNRKARAKRKQQRLPQKEGESEVDTDGESSREKKPKIDDEFNKED
AGYSEKNVDAFEKRHGIFLMGSAQTEAIPQIHERSMNNIGLRHADTEAQGFEKNHGVINQ
KTHANDQQQFNGTRENVSSSLLHFQTEADLNIDTSLERTGNAAIVSTENDQRGSRVMTVL
LDGKQFEVPVGVVDVRRTFGDTAVLLDSHGHVVPTNDSGMTFHPLHGSESYTLLR

>PmWOX2

MPSVTQGSAMANPNFVALHNNQVMTEEQMETLRRQICVYSTICSQLVEMHRAMSQQQP
SSVPTLLMGQHMLYDLAQGNPGFRTSARQRWTPSQTQLQILESLFDQGHATPSKQKIKEI
TMELSQHGQISETNVYNWFQNRKARAKRKQLPSQRGGESENETDDEYPGEKRFKPQHDS
NAQNPKSGHSEADPQVHKSDDVVQNKPCTTDQKQFGIQENDTQSLLPREDEMRPHASLG
STMLGVSGSAEHESVECLPEVKPIYSSVEVGNRDHDATQAYIHGSGTTTGQVDGKSWQV
PSVVVDVRRMFGENAVLVDSRGHMVPTNDMGVSFHPLQASEGYTVLSQYQCASGVPVH
ALRANTGDPYRGT

>PmWOX3

MEAKMANLNSVTNMPFACENGGCNKRFRPLVPKLTLSPNSAAPMANAVWSTQQSHDQ
YSQLQQAFSSATSGNSVSVSAAACLLEPKISRLDGKVDDNSNQRNQIDRLIKGDQQNMKE
EQAESCAVQSQPAASGTRWNPTPDQIRILEMFYKGGMRTPNAEQIEHITAQLRQYGKIEG
KNVFYWFQNHKARERQKQKRNSSMHQVAATAAKKTPTTIMADNPNELHKPNSNGTYSL
YNLPFTAMSEDSRLMFTSTSLLTMPCSKGLAASMAQVGVDVLTPATTGAKRDGLDVLKR
SSHGPPQVQVNEEGSPSKNSMIWDSINSSGEGIITNLALSQVAADMPVNAVNSCKRKNRL
WQVMSPNEELGLHTESSSESICLQTSNSDPINPSDYTNEEDHRQLQTLQLFPLRPEGSLKSK
GETNMDNSVLSLRVNPSSTYPFREFNKSAESSTQMGAAIFEEHKYSKAAEFFPNLSADYSF
LNSMT

>PmWOX4

MTMEMNSPNIIADMQGIGLRGVGSQHPLSFTMQEQVMTEEQLETLRRQISVYATICQQLV
HMHRAVVLQMDPMRGMPLGQAIAFDSLMGMAGQRVTARQRWTPSQVQLQILEKIFDQ
DKGTPSKQKIKEITAELSQHGHISETNVYNWFQNRRARTKRKQQIVTSNSGESEMDNSLD
SSKGKQVKMDETIVQETQLLRQSSVAFQGHENVPKQQKDDTQQCRSAVSFSSQASSNALI
DFAKNASFEQGFPETRNGQLMDFDKPMENSNTISTGEGYIMIGNQAWLQSDPIKRFP

>PmWOX5

WQGFMVCNTNDGQLIELSNVYIRPSWAYREGIRNPTPDQINEIAGRLRTYGAIQSKSVYF
WFQNRKTAERRQKEERVSPINQFLQPHSFAGTSHRNASKDDSEGLNAGNSGERRTLELFP
VHPTNVQEYGSHEPATSLWFTITLYIRQRILNLERDLSVKASDGLSGELRT

>PmWOX6

GQEGEIAQPKEKLRWIPKPEQIRILEAMFNSRIVNPPREQIKTMRARLEQFGEVHDTNVFY
WFQNRKSKIKRRQRQPQAAQRYNSSTETGNPSSSLSMSTTSTSDQSFLDLQLVRSA

>PmWOX7

DEEKNMPRSVTAKWKPTTEQRTILEYIYRSGLRTPKAEEIERITVDLKKYGNVEGKNVFY
WFQNFNARERHQRRNFTGDE

>PmWOX8

QLQILENIFDQGNGTPSKQKIKEITAELTQHGQISETNVYNWFQNRRARTKRKQQLGLSN
NGESEVETDLESPKEKKAKMDHNPIRETPVARQTDMSFPCSDQHHFESQQ

>PmWOX9

MNDIKGSGETSSGGYYYTLIYFLQDYERLFCCIEFNGVVSRARLRVPTAMPQPATTRWNP
TPEQLMILEEMYRGGIRTPNADQIQQITAHLALYGKIEGKNVFYWFQNHKARDRQKLRR
KMESKQEPKRSTFEDESALNINNGWRLSTLESREGCCNSFNCGSVEEYDCEIDTVHAISRR
ARPLKTLEL

>PmWUS

MESAERIGSYDVYRQPSSTRWNPTAEQLSILRELYYTNGIRSPTVDEIHRISMKLSRYGKIE
GKNVFYWFQNHKARHRQKTRLNAMNVAAFPTFHTKSFYEKRMVGENSSCEEAACCSLE
QMYAVKHHDVMKAMEENQLQEGKINRHVETLELFPTHCEDRSPEPSEKKSCCWGSCET
KENHNNNPPGGYKGKQRDSVLDLCLTLGNRSCGLHDN

>PmWOXX

MSSIPGGRETRRCSPREQLRILEAIYNGGNQTPKPEQIQVIAAELRRHGTVAGINVFYWFK
NRKARERRKSRSIQEAHAAANSSGFSSFQPNDHFSSEMTVGETNYLLNSNSEEDESKFSNC
VEVYDFLLNSDSAAAEVGVEVESSLQTLQLFPPSCTESMAYNNHQQ

Table S3. Accession numbers (GenBank) of all members in phylogenetic analysis .

Protein ID	Accession
GbWOX2	CAT02902
GbWOX3A	CAT02903
GbWOX3B	CAT02904
GbWOX4	CCP29680
GbWOX9	CCP29683
GbWOX13	CCP29682
GbWUS	CAT02906
PaWOX2	CAL18267
PaWOX3	AGL53581
PaWOX4	AGL53582
PaWOX5	AGL53583
PaWOX8/9	ADF42580
PaWOX8A	AGL53584
PaWOX8B	AGL53585
PaWOX8C	AGL53586
PaWOX8D	AGL53587
PaWOXG	MG545153/AUJ87558.1
PaWOXX	ANC94893
PaWUS	AGL54197
PpWOX2	ANC94872
PpWOX3	ANC94873
PpWOX4	ANC94874
PpWOX5	ALN42234
PpWOX13	ANC94875

PpWOXA	ANC94877
PpWOXB	ANC94878
PpWOXC	ANC94879
PpWOXD	ANC94880
PpWOXE	ANC94881
PpWOXF	ANC94882
PpWOXG	MG545154
PpWOXX	ANC94876
PpWUS	ALN42231
PsWOX2	CAT02937
PsWOX3	CAT02936
PsWOX4	CCP29681
PsWUS	CAT02938
PtaWOX2	ANC94883
PtaWOX3	ANC94884
PtaWOX4	ANC94885
PtaWOX5	ANC94886
PtaWOX13	ANC94887
PtaWOXA	ANC94889
PtaWOXB	ANC94890
PtaWOXE	ANC94891
PtaWOXG	MG545155
PtaWOXX	ANC94888
PtaWUS	ANC94892
PtWOX1	Pt2G51570
PtWOX2	Pt0G26920
PtWOX3	PtJG03550
PtWOX5	PtXG04970
PtWOX6	Pt8G58080
PtWOX7	PtQG16880
PtWOX10	Pt8G00270
PtWOX12	Pt5G40070
PtPRS1	Pt3G38920
PtPRS2	Pt3G36820
PtWUS	Pt3G38940
PtPFS2	Pt6G50390
OsWOX3A	XP_015619605
OsWOX3	BAE48302

OsWOX2	NP_001044759
OsWOX4	CAJ84142
OsWOX5	NP_001044837
OsWOX7	Q0JKK6
OsWOX9C	EEE55148
OsWOX9D	NP_001174527
OsWOX10	A2YST1
OsWOX11	XP_015645719
OsWOX6	XP_015629006
OsWOX13	NP_001044631
OsWUS	BAE48303
ZmNS1	CAD60454
ZmNS2	NP_001105242
ZmWOX5	NP_001306689
ZmWOX5B	XP_008657078
ZmWOX3A	CAM32346
ZmWOX3B	CAM33396
ZmWOX4	CAM32347
ZmWOX5A	CAM32348
ZmWOX9	NP_001306687
ZmWOX9A	CAM32350
ZmWOX7	NP_001288414
ZmWOX12	NP_001288483
ZmWOX11	NP_001147238
ZmWOX11A	XP_008653489
ZmWOX12B	ACR38139
ZmWOX13A	DAA44071
ZmWOX8B	XP_008657129
ZmWOX8	NP_001288430
ZmWOX14	ACG33593
ZmWUS1	CAJ84136
ZmWUS2	NP_001105961
PtrWOX1A	XP_002317877
PtrWOX1B	XP_011008807
PtrWOX2	XP_002298364
PtrWOX4	XP_011029533
PtrWOX5A	XP_002312145
PtrWOX5B	XP_002315173
PtrWOX6	XP_002314802
PtrWOX9	XP_002305099

PtrWOX11	XP_002325442
PtrWOX12	XP_002319197
PtrWOX13	XP_002306432
PtrWUS	CAJ84139
AtWOX1	AAP37133
AtWOX2	AAP37131
AtWOX3	AAP37135
AtWOX4	AAP37134
AtWOX5	AAP37136
AtWOX6	AAP37137
AtWOX7	NP_196196
AtWOX8	AAP37138
AtWOX9	AAP37139
AtWOX10	NP_173494
AtWOX11	NP_001118563
AtWOX12	AAP37141
AtWOX13	AAP37142
AtWOX14	NP_173493
